# Older adults’ experiences of adapting to changes after colorectal cancer surgery in Sweden: a qualitative study

**DOI:** 10.1136/bmjopen-2026-124929

**Published:** 2026-10-12

**Authors:** Anahita Amirpour, Amina Guenna Holmgren, Ulrica Nilsson, Jeanette Eckerblad

**Affiliations:** 1 Neurobiology, Care Sciences and Society, Karolinska Institutet, Stockholm, Sweden

**Keywords:** SURGERY, ONCOLOGY, GERIATRIC MEDICINE

## Abstract

**Objectives:**

To explore older adults’ experiences of adapting to physical, emotional and cognitive changes during the first month after colorectal cancer surgery.

**Design, setting, participants:**

A qualitative descriptive study with an inductive approach was conducted in the Stockholm region, Sweden. Thirteen adults aged ≥60 years who had undergone planned colorectal cancer surgery participated in semi-structured interviews 1-month postoperatively. Data were analysed using thematic analysis following Braun and Clarke’s guidelines.

**Results:**

Participants described recovery as a gradual and multifaceted process shaped by changes in their physical, cognitive and emotional capacities. Everyday activities required deliberate planning, pacing and continuous adjustment to fluctuating energy and function. These adaptations took place within a relational context, where support from family and healthcare professionals was experienced as both enabling and demanding. A heightened awareness of mortality emerged following the cancer diagnosis and remained present in daily life, influencing how participants prioritised and engaged in activities. Over time, participants navigated uncertainty through efforts to balance management and acceptance while striving to maintain autonomy and involvement in daily life.

**Conclusions:**

Recovery after colorectal cancer surgery in older adults is a dynamic and multidimensional process shaped by altered bodily capacity and awareness of mortality. Rehabilitation should extend beyond physical function to support everyday adaptation, autonomy and patients’ capacity to navigate uncertainty during the postoperative period.

**Trial registration number:**

NCT05564195.

**STRENGTHS AND LIMITATIONS OF THIS STUDY**

- The study provides in-depth accounts of older adults’ experiences during the relatively underexplored first month after colorectal cancer surgery.
- Although information power was considered sufficient, the small homogenous group of Swedish-speaking, highly educated participants may limit transferability of thefindings

## Background

 Colorectal cancer (CRC) is the third most common cancer type in the world and the second most common type in Europe in both sexes,^1^ primarily affecting individuals 65 years or older.^2^ Treatment usually involves surgery, alone or in combination with radiation and/or chemotherapy.^2^ Advances in screening programmes, surgical techniques and systemic therapies have substantially improved survival rates.^2^ As a result, an increasing number of individuals are living longer after CRC treatment, highlighting the importance of understanding survivorship experiences following surgery.^3^

While improved survival reflects progress in CRC management, treatment can have significant and lasting effects on individuals’ lives. Physical, emotional and social functioning can be altered with consequences in daily lives and overall well-being.^4 5^ Patients often face physical side effects, functional limitations and uncertainty about the future, all of which can challenge their ability to resume everyday activities and social roles.^6^ During and after treatment, patients can experience multiple concurrent symptoms, including gastrointestinal dysfunction as well as psychological and cognitive symptoms such as anxiety, body image distress and fatigue,^7^ which intersect and shape how they manage everyday life after being discharged home.

Previous qualitative research has explored different aspects of patients’ experiences following colorectal surgery, including physical recovery,^8^ preparedness for surgery,^9^ living with an ostomy^4^ and the broader treatment journey from diagnosis to treatment.^5 10^ However, most studies have focused on either isolated aspects of recovery or the overall treatment journey of care, rather than the first month postoperatively. In particular, little is known about how older adults adapt to the physical, emotional and cognitive changes that occur during the first month after surgery.

To address this gap, this study aimed to explore older adults’ experiences of adapting to physical, emotional and cognitive changes during the first month after CRC surgery.

## Methods

### Study design

A qualitative descriptive approach^11^ was used to explore older adults’ experiences of physical, emotional and cognitive changes 1 month after CRC surgery. The current study was conducted alongside a larger feasibility trial previously published,^12^ where the aim was to evaluate the usability of a digital, cognitive test battery in older adults undergoing abdominal surgery. Reporting of study findings adheres to the consolidated criteria for reporting qualitative research, COREQ (online supplemental file 2).^13^

### Setting and participants

The study was conducted in the Stockholm region, Sweden, where participants were recruited face-to-face by the hospital’s research staff. The research staff provided study information and obtained written informed consent from the participants. Using a purposive sampling approach, 13 participants, aged ≥60 years, who had undergone planned CRC surgery lasting ≥60 min, were included. Semi-structured interviews were conducted 1 month after surgery. Exclusion criteria included preoperative Mini-Mental State Examination (MMSE) score <23, current psychiatric or central nervous system illness, uncorrected sensory or motor impairments, defective colour vision or insufficient proficiency in Swedish. The Swedish version of the MMSE was used with written permission from Psychological Assessment Resources. Participant-related characteristics are presented in table 1.

**Table 1:** Summary of participant characteristics

Age in years, range (median)	63–90 (80)
Sex, n	
Women	5
Men	8
Education level, n	
University	6
Post-secondary	2
Upper secondary	3
Elementary	2
Preoperative MMSE score, median	29
Postoperative physical complications*, number of participants	5

* Type of postoperative physical complications were mainly surgical, for example, wound infection and one non-surgical (kidney infection).

MMSE, Mini-Mental State Examination.

### Data collection

Data collection took place from March 2023 to June 2024. Participant characteristics were obtained through case report forms at the hospital. All 13 interviews were conducted by the first author, AA, and took place face-to-face in the participant’s home, at the premises of Karolinska Institutet or virtually via Zoom. Interviews were conducted one-to-one.

The interviews were performed with the help of an interview guide (online supplemental file 1) with open-ended questions focusing on cognitive and emotional changes after the surgery. Examples of questions were “Can you tell me how you’ve been since you were discharged from the hospital?” and “How has your mood been after your surgery?”. The interview guide was pilot tested in an interview, and a new question was added (Q3, online supplemental file 1). The pilot interview is included in the findings. Guided by the notion of information power,^14^ data collection proceeded with an ongoing assessment of the richness and relevance of the interview material. Interviews continued until the data were judged to provide sufficient depth and breadth to address the study’s aim. The interviews were audio-recorded, and interview length ranged from 27 to 60 min, with a median time of 38 min. The interviews were transcribed verbatim by a transcription service company.

### Data analysis

The analysis was guided by Braun and Clarke’s guidelines for thematic analysis, following the six-phase process.^15^ The analysis was conducted at a semantic level and followed an inductive approach, allowing themes to be derived from the data rather than a pre-existing theoretical framework.^16^

Authors AA, AGH and JE familiarised themselves with the data through repeated reading of the transcripts, repeated listening to the interviews and making initial notes. AA produced written reflections after reading and listening to each transcript. Initial manual coding was independently undertaken by AA, AGH and JE across the dataset, focusing on participants’ descriptions relevant to the study’s aim. The authors engaged in ongoing discussions about their interpretations and the development of codes, and the written reflections supported the subsequent development of themes. Once initial coding was completed, AA and JE searched for patterns of shared meaning across the dataset and organised the codes into preliminary themes and subthemes. The illustrative quotes were translated from Swedish to English by AA and JE. An example of the analytical process is presented in table 2.

**Table 2:** Example of analytical process

Codes	Subtheme	Theme
Vigilance, pain and pain relief, energy, fatigue, endurance, complications and setbacks	Adapting to altered bodily capacity	Relearning everyday life in a changed body

### Rigor and reflexivity

To enhance trustworthiness, multiple researchers independently analysed the data before comparing and discussing their interpretations, allowing different perspectives to inform and challenge the analysis. The purpose was not to reach consensus, but to critically examine how different interpretations and researcher perspectives shaped the analysis. All authors were then involved in reviewing and refining the codes, themes and subthemes through an iterative and reflective process, continuously discussing preunderstandings and how the researchers’ perspectives could shape the analysis.

Dependability was supported by using a consistent interview guide, having all interviews conducted by the same researcher and adherence to the analytic process. Transferability was facilitated by providing detailed descriptions of the participants, data collection and analysis. In thematic analysis, the researcher is considered an integral part of the research process, and their perspectives and positioning inevitably influence the analysis and interpretation. To enhance transparency and reflexivity, the researchers’ backgrounds are therefore described.^16^ All authors are registered nurses with PhD degrees, with clinical experience in perioperative care and expertise in qualitative research. None of the authors were involved in the care of the participants. The research team’s background in perioperative care may have influenced which aspects of participants’ experiences were explored during the interviews and may have shaped which aspects of the data were attended to and interpreted as meaningful during the analysis. Familiarity with perioperative care provided contextual understanding but may also have introduced assumptions about recovery and adaptations after surgery.

## Results

### Relearning everyday life in a changed body

The themes and sub-themes are presented in table 3. Life after discharge was experienced as a process of relearning how to manage everyday life in a body that could no longer be taken for granted. Activities that had previously been routine, such as walking, household chores or social engagement now required planning, pacing and ongoing vigilance to bodily signals.

**Table 3:** Themes and sub-themes of the result

Themes	Sub-themes
Relearning everyday life in a changed body	Adapting to altered bodily capacity
	Managing emotional and cognitive changes
	Rebuilding autonomy through supported self-management
The fragility of life and the presence of death	Intrinsic adaptation through confronting mortality
	Belief in a just world
	Struggle for control and autonomy

#### Adapting to altered bodily capacity

Relearning everyday life was experienced as an active and ongoing process, where participants gradually reestablished routines, regained confidence in their bodies and reoriented themselves toward future possibilities. Central to this process was becoming attentive to bodily signals and adapting activities to match current capacities, which contributed to a growing sense of security and independence.

After returning home, the demands of everyday life often revealed physical limitations that had not been apparent at the hospital or rehabilitation setting. As a result, activities required deliberate energy management. Participants described using flexible strategies, such as pacing themselves, dividing tasks into manageable steps, postponing or limiting activities and asking for help when needed. These adjustments were not perceived as setbacks but as necessary and constructive ways of continuing to engage in everyday life.

Goals related to work, travel, physical activity or personal interests served as motivators and helped participants focus on possibilities rather than limitations. For some, bodily changes also affected self-perception. One participant who had received an ostomy described distress related to his altered body image and feelings of shame, showing how bodily changes influenced his identity and self-image.

[…] it was an unpleasant feeling and a fear that I might wake up at night and… well… find the bed soiled. That’s not something you think is very nice when you’re over 60. You feel like you should be able to manage your bowel movements somehow. But that’s gone now. At first, it was the scars […] And then there were the practical problems with the leaking bag. And also, the thought that… I mean, I’m not the kind of person who thinks, oh, I’m going to stand on the beach and show off like Tarzan with a six-pack. But it’s still not very attractive to have a bag hanging on your belly. So, there’s a bit of a sense of shame there somewhere, which affects you as well (Participant 17, man).

#### Managing emotional and cognitive changes

Some participants described having difficulties with recalling recent events and maintaining focus. Mental fatigue was common. These changes required participants to actively regulate their daily activities and adjust expectations regarding what they could manage. To cope with these disruptions, participants used written reminders, calendars and structuring their days, while also allowing flexibility by adjusting activities according to their daily condition. Some attributed these changes to ageing, whereas others experienced them as new and unexpected limitations following surgery. In addition to cognitive changes, participants described increased emotional reactivity, including irritability, a lower tolerance for stress and stronger responses to minor frustrations in everyday situations. These reactions were often described as disproportionate compared with before surgery and required conscious effort to manage. Strategies such as taking walks, seeking distraction or spending time with pets or family members were used to regulate these emotional responses.

Before each (cancer) result, these three times, it (the anxiety) lasts for a couple of hours. So, it hits immediately. Poff, and the anxiety strikes. And then, somehow, whether I process it or suppress it, I don’t know, but it’s gone after just a few hours. And then you just go through the treatments, and then it’s done. After that, I don’t worry at all (Participant 14, woman)

Participants varied in how they managed periods of heightened emotional strain. For some, anxiety responses were intense but short-lived, allowing them to return relatively quickly to daily activities. For others, low mood was more persistent and closely linked to situational factors, such as postoperative complications or the hospital environment. Experiences of inactivity, loss of control and repeated medical interventions contributed to feelings of distress during these periods; one participant described feeling like a ‘rat’ during hospitalisation.

#### Rebuilding autonomy through supported self-management

Relearning everyday life was also shaped through interactions with healthcare professionals and participants’ social networks. Healthcare professionals provided guidance, reassurance and follow-up after discharge, supporting the transition from a structured hospital environment to more independent management at home. This shift required participants to assume greater personal responsibility and integrate professional advice into their daily routines. Access to rehabilitation services and positive feedback at the follow-up appointments supported a gradual increase in their autonomy, while reinforcing a sense of trust in their own capabilities. This also impacted their perception that recovery was going effectively.

I feel a great sense of security in what they have done and with the care, I must say, that the staff has shown me. Uh, they have been understanding, and they have explained. They have even contacted me after I was discharged to check how I’m doing once I got home. (Participant 23, man)

Support from family, friends and wider social networks was also described as essential for managing both practical and emotional challenges. Participants adjusted their level of social engagement according to their energy and physical capacity. For some, this involved drawing firmer boundaries and limiting participation in larger gatherings while maintaining contact through smaller interactions or phone calls. Others experienced social interaction as energising, although it could still lead to increased fatigue.

I notice it, when I come home from… after that period… you meet family and children and grandchildren and all of that. I get tired, you know. I get more tired than I did before the surgery. I notice that. And I feel a bit dull, like. I don’t really want to join in. I don’t want to bring the mood down […] Everyone is very caring and warm and nice, and they want you to be involved. But I can also feel like, no, today I want to take a step back. That’s how I can feel. (Participant 20, man).

### The fragility of life and the presence of death

Patients’ existential reflections extended beyond death itself to include how illness impacts experiences of meaning, justice, autonomy and dignity. A cancer diagnosis prompted existential considerations regarding life’s finitude, fairness and purpose. Thoughts of death were intermittent, such as expressing fear of dying, thoughts around suffering, loss of autonomy and achieving a dignified end to life.

### Intrinsic adaptation through confronting mortality

A cancer diagnosis often served as an existential turning point, making the finiteness of life both tangible and unavoidable. For several patients, thoughts of death emerged immediately on receiving the diagnosis and were perceived as a shock. In some cases, this led to early practical considerations regarding end-of-life planning.

It has meant that the initial shock, or the sort of confinement within the illness, has gradually disappeared. In the beginning, it was quite intense. I cancelled all my work tasks, well, all my additional tasks. We turned down some meetings we had, or dinners and other plans we had with friends, immediately. But after some time, when I had processed everything, understood what it was about, and reflected on it, that feeling also eased. But initially it was very… a very tough… yes, you could almost call it a shock. It was quite hard to take in initially. (Participant 23, man)

For others, awareness of death was less tied to fear and more to reflections on ageing, dementia and a dignified end of life. Prior experiences of cancer, death and loss played a central role. The loss of family members or friends shaped how participants interpreted their own situation, contributing both to heightened vulnerability and to a more accepting and positive outlook. Despite facing a serious diagnosis, many expressed gratitude for the care they received and for the opportunity to continue living. One participant reflected that the worst had already happened after losing both a spouse and a grandchild, which appeared to lessen their fear of their own mortality.

Attention instead shifted toward concerns about prolonged suffering, with a preference for dying in good health rather than enduring severe pain or losing autonomy due to conditions such as dementia. Death thus emerged not only as a threat but also as something requiring practical and dignified engagement. This was reflected in participants’ considerations of assisted dying and their desire to avoid becoming a passive, dependent person confined to the home, demonstrating how awareness of mortality can lead to concrete existential decision-making.

#### Belief in a just world

A recurring theme in participants’ narratives was a profound sense of life’s unfairness. The participants described feelings of shock, confusion and frustration on receiving their cancer diagnosis, particularly because they believed they had lived healthy, responsible and risk-averse lives. This perceived mismatch between effort and outcome contributed to a broader questioning of justice and predictability in life.

Patients also engaged in efforts to find causes and meaning in their illness. This included reflections on whether lifestyle choices might have contributed to disease onset or whether specific dietary choices (eg, consuming mushrooms) could provide an explanation.

After my husband died of cancer… I never thought he would, he was so… he was so healthy and, you know… We lived quite properly… I thought we were very proper people. He hadn’t been careless with his diet or… well, anything like that. So, the fact that he got cancer didn’t feel fair for me… And then I’ve also thought, well, maybe it’s because I’ve made too many mushroom stews. Yes, we like going out into the forest to pick mushrooms, maybe that’s what isn’t good. And then I thought I might get it too. (Participant 2, woman)

Such reasoning can be understood as an attempt to create coherence in an otherwise chaotic and threatening situation. At the same time, participants recognised the limits of these explanations contributing to a gradual acceptance of uncertainty. For some, the perceived injustice of illness was heightened by the timing of the diagnosis. Receiving a cancer diagnosis just before Christmas, a holiday already imbued with grief from previous losses, linked the disease to existential reflections on life, death and tradition.

In some cases, participants balanced this sense of unfairness with more hopeful perspectives, such as describing themselves as a “Sunday child”, a symbol of luck and resilience despite life’s inexplicability.

#### Struggle for control and autonomy

The participants articulated a pragmatic orientation toward their circumstances, expressing the view that events would unfold regardless of their wishes and that investing emotional energy in what could not be changed felt futile. Within this context, control was redefined as an internal capacity to choose one’s attitude toward uncertainty rather than influence outcomes. Fear of losing control over one’s body and life emerged as a central existential concern. Anxiety surrounding the surgery and altered bowel function was not merely physical but also represented threats to independence and dignity. Participants described feeling confined at home due to gastrointestinal issues, illustrating how bodily limitations quickly translate into existential constraints, reducing freedom and life space. This highlights the embodied nature of existential distress. The fear of dependence extended into future considerations. Concerns about becoming care-dependent, losing decision-making capacity or developing dementia created a need to reclaim control.

Participants described engaging in a range of practical, future-oriented tasks, such as drafting wills, organising legal and financial documents and planning daily life in careful detail. These activities served as strategies to preserve a sense of autonomy and to prevent becoming a burden to others. Alongside these efforts to maintain control, a parallel process of acceptance emerged, as participants acknowledged the limits of what could be managed and instead focused on adapting to their circumstances. Participants acknowledged the inherent uncertainty of their condition and the impossibility of achieving full reassurance regarding cancer recurrence. In response to this uncertainty, some participants assumed a fatalistic approach. They gradually shifted toward acceptance. They recognised that although cancer made life feel more fragile, it did not necessarily imply immediate death. This perspective allowed them to focus on the present moment and prioritise meaningful activities. Here, control was less about dictating future outcomes and more about choosing one’s attitude toward uncertainty.

No, but you know, when you’re worrying…I guess it’s when you get cancer, you wonder what it really means. Am I going to die now, or… Yes. Eventually, you realize that you’re not going to die from this tomorrow. It takes a while. And who the hell is in a hurry? […] But you also realize that there’s nothing you can do. It will be what it will be. The journey there. It’s better if it’s at least somewhat enjoyable, than…than going around feeling depressed about it. […] So, I’m a bit of a fatalist, like that. (Participant 18, man)

## Discussion

Recovery during the first postoperative month involved more than physical healing and was experienced as an ongoing reconfiguration of everyday life in the context of a changed body and heightened awareness of mortality. The participants became acutely aware of their altered bodily capacities as they attempted to restore continuity in their daily lives. The experience of no longer being able to perform certain tasks or keep up with usual routines, described by participants in the study by Wan *et al*^17^ as ‘crippling’, highlighted the profound disruption to their sense of self and independence. Similarly, as shown in the study by Keny *et al*^18^ older adults who had undergone major surgery experienced at-home restrictions that limited their ability to engage in valued daily activities, such as driving, exercising and socialising, and these limitations further shaped their overall recovery experience.^18^ Yet, over time, participants began to re-establish routines, regain confidence in their bodies and reorient themselves toward future possibilities, findings that are consistent with previous research.^6 17 19–22^ Central to this process was developing attentiveness to bodily signals and adapting activities to match current capacities, which contributed to a growing sense of security, independence and acceptance. Control thus shifted from shaping outcomes to shaping one’s response to uncertainty, an adaptive process consistent with resilience research, which conceptualises resilience as the dynamic maintenance or re-establishment of functioning in the face of adversity.^23 24^

We also found that participants experienced difficulties recalling recent events and maintaining focus, which some attributed to age-related cognitive changes. Older age is a well-established risk factor for postoperative cognitive impairment, in which multiple cognitive domains may be affected, including memory, attention, information processing and executive functioning.^25^ Additionally, research indicates that older adults with advanced cancer who exhibit impaired cognitive function have a higher prevalence of other health-related challenges, including reduced physical functioning, depression and anxiety.^26^ In line with previous research,^27–29^ we also found that participants experienced notable emotional changes. It has been reported that patients undergoing colorectal surgery tend to adopt more emotionally focused coping strategies, whereas their next of kin are more likely to engage in problem-focused approaches.^17^ Moreover, unexpected emotional and behavioural changes may heighten anxiety and fear among next of kin, particularly when they struggle to recognise their family member, who appears unfamiliar as a consequence of these changes.^25^

Alongside these embodied adjustments, recovery was shaped by a heightened awareness of mortality following the cancer diagnosis. Participants described how thoughts of death arose on diagnosis and were experienced as disruptive and unavoidable. Although prior research has documented fear of cancer recurrence, loss of identity and feelings of powerlessness,^6 22 30^ our findings show how these existential concerns remain present during early recovery and are interwoven with everyday life. Over time, participants described a shift towards a more pragmatic orientation, characterised by acceptance, present-focused attention and efforts to maintain meaningful engagement in daily life. This process can be understood as a form of meaning-making, where individuals integrate serious illness into their broader life narrative.^23 24^ Cancer can profoundly disrupt daily life, making spirituality an important coping resource.^31^ Spirituality is commonly understood as a personal search for meaning, purpose, hope and inner peace and may provide strength beyond one’s immediate capacities.^32^ For older adults, strong spiritual beliefs can be especially valuable in managing chronic illness and sustaining life satisfaction. When coupled with a sense of hope, spirituality may support resilience in facing the challenges associated with cancer.^23 31 33^ Among older adults, spirituality has been linked to successful ageing, enhanced coping with serious illness or injury and more positive health outcomes.^34^ Research also demonstrates a positive association between higher levels of spirituality and improved quality of life among cancer survivors.^35^

In contrast to findings in other settings, participants in our study did not describe financial burdens related to treatment or access to care.^5 17 22^ This may reflect the Swedish healthcare system, which is predominantly tax-funded and provides universal coverage.^36^ The urban context of the study may also have contributed to this finding, as participants lived in a region with relatively short travel distances to specialised care. These contextual factors may have reduced external stressors during recovery and should be considered when interpreting the findings.

Taken together, these findings suggest that recovery after CRC surgery in older adults involves a continuous interplay between adaptation to a changed body and confrontation with existential uncertainty. Participants’ strategies, ranging from active adjustment and planning to acceptance and letting go of control, reflect a dynamic process of maintaining and re-establishing functioning under conditions of vulnerability. This aligns with conceptualisations of resilience as an ongoing and context-dependent process, rather than a stable individual trait.^23 24^ However, our findings also indicate that this process involves periods of instability, emotional and cognitive strain and identity disruption, highlighting the need to understand recovery as fluctuating and multidimensional.

### Strengths and limitations

This study has some strengths. The interviews provided rich, in-depth accounts of older adults’ experiences during the first postoperative month, a period that remains relatively underexplored in CRC recovery. Conducting interviews 1 month after surgery allowed participants to reflect on their transition from hospital to home, capturing both immediate challenges and early processes of adaptation. All interviews were conducted by the same researcher, ensuring consistency in data collection and supporting the depth and coherence of the material. In addition, the involvement of multiple researchers with expertise in qualitative methods strengthened the analytic process through ongoing discussions and reflexive engagement with the data. This facilitated a nuanced interpretation of the findings.

Some limitations must be acknowledged. The sample was relatively small; however, guided by the principle of information power,^14^ the data were considered sufficient to address the study aim. The homogeneity of the sample, with participants being Swedish-speaking and highly educated, may have limited the range of perspectives captured and influenced the variation in experiences. Previous research suggests that individuals with higher educational levels may respond differently to a cancer diagnosis^17^ which should be considered when interpreting the findings. Future studies including more diverse populations would enhance the breadth and applicability of the findings.

## Conclusions

Recovery after CRC surgery in older adults is an ongoing reconfiguration of everyday life shaped by both bodily changes and awareness of mortality. Participants actively adapted to changing physical, cognitive and emotional capacities through continuous adjustment of routines, expectations and engagement in daily activities. This process unfolded within a relational context and was characterised by efforts in acceptance of uncertainty. These findings highlight recovery as a dynamic and multidimensional process, and rehabilitation should therefore move beyond a sole focus on physical function to support patients’ everyday adaptation, autonomy and capacity to navigate uncertainty during the postoperative period.

## Supplementary material

10.1136/bmjopen-2026-124929 online supplemental file 1

10.1136/bmjopen-2026-124929 online supplemental file 2

## Data Availability

No data are available.

## References

[1] Bray F, Laversanne M, Sung H (2024). Global cancer statistics 2022: GLOBOCAN estimates of incidence and mortality worldwide for 36 cancers in 185 countries. CA Cancer J Clin.

[2] Wagle NS, Nogueira L, Devasia TP (2025). Cancer treatment and survivorship statistics, 2025. CA Cancer J Clin.

[3] Langbaum T, Smith TJ (2019). Time to Study Metastatic-Cancer Survivorship. N Engl J Med.

[4] Asefa T, Endale HT, Getnet M (2025). The impact of ostomy on colorectal cancer patients and caregivers: a qualitative study. Support Care Cancer.

[5] Erdoğan Yüce G, Döner A, Muz G (2025). Exploring the Experiences and Needs of Patients With Colorectal Cancer: A Qualitative Study. Gastroenterol Nurs.

[6] Lim CYS, Laidsaar-Powell RC, Young JM (2022). The long haul: Lived experiences of survivors following different treatments for advanced colorectal cancer: A qualitative study. Eur J Oncol Nurs.

[7] Han CJ, Yang GS, Syrjala K (2020). Symptom Experiences in Colorectal Cancer Survivors After Cancer Treatments: A Systematic Review and Meta-analysis. Cancer Nurs.

[8] Jakobsson J, Idvall E, Kumlien C (2017). The lived experience of recovery during the first 6 months after colorectal cancer surgery. J Clin Nurs.

[9] Cuijpers ACM, Lubbers T, van Rens HA (2022). The patient perspective on the preoperative colorectal cancer care pathway and preparedness for surgery and postoperative recovery-a qualitative interview study. J Surg Oncol.

[10] Hannah C, Eriksson LE, Ramwell A (2025). Experiences and perspectives of patients undergoing colorectal cancer surgery: A qualitative study. Eur J Oncol Nurs.

[11] Sandelowski M (2000). Whatever happened to qualitative description?. Res Nurs Health.

[12] Amirpour A, Saarijärvi M, Eckerblad J (2025). Evaluation of a Digital, Self-Administered, Cognitive Test Battery in Older Adult Patients Undergoing Abdominal Surgery: Nonrandomized Feasibility Trial. JMIR Form Res.

[13] Tong A, Sainsbury P, Craig J (2007). Consolidated criteria for reporting qualitative research (COREQ): a 32-item checklist for interviews and focus groups. Int J Qual Health Care.

[14] Malterud K, Siersma VD, Guassora AD (2016). Sample Size in Qualitative Interview Studies: Guided by Information Power. Qual Health Res.

[15] Braun V, Clarke V (2006). Using thematic analysis in psychology. Qual Res Psychol.

[16] Braun V, Clarke V (2022). Thematic Analysis: A Practical Guide.

[17] Wan SW, Chong CS, Jee XP (2022). Perioperative experiences and needs of patients who undergo colorectal cancer surgery and their family caregivers: a qualitative study. Support Care Cancer.

[18] Keny C, Dillon EC, Russell MM (2024). “It’s Incapacitated Me in so Many Ways”: Older Adults’ Lived Experience With Postoperative Symptoms at Home After Major Elective Surgery. Ann Surg.

[19] Andersson TK, Engström M, Bjerså K (2022). Perceptions of Experiences of Recovery After Pancreaticoduodenectomy-A Phenomenographic Interview Study. Cancer Nurs.

[20] Nilsson U, Jaensson M, Hugelius K (2020). A journey to a new stable state-further development of the postoperative recovery concept from day surgical perspective: a qualitative study. BMJ Open.

[21] Lundmark M, Lennerling A, Almgren M (2016). Recovery after lung transplantation from a patient perspective - proposing a new framework. J Adv Nurs.

[22] Lim CYS, Laidsaar-Powell RC, Young JM (2021). Colorectal cancer survivorship: A systematic review and thematic synthesis of qualitative research. Eur J Cancer Care (Engl).

[23] Seiler A, Jenewein J (2019). Resilience in Cancer Patients. Front Psychiatry.

[24] Overcash J, List AF, Balducci L (2023). Resilience in Older Adults Diagnosed With Cancer and Receiving Chemotherapy. Clin J Oncol Nurs.

[25] Nilsson U, Amirpour A, Lampi M (2025). Older Patients’ Postoperative Neurocognitive Recovery: A Narrative Review. Clin Interv Aging.

[26] Kyi K, Consagra W, Culakova E (2024). Comparing Mini-Cog and Blessed Orientation-Memory-Concentration test for evaluating cognition in older patients with advanced cancer. J Geriatr Oncol.

[27] Amirpour A, Bergman L, Markovic G (2025). Understanding neurocognitive recovery in older adults after total hip arthroplasty-neurocognitive assessment, blood biomarkers and patient experiences: a mixed-methods study. BMJ Open.

[28] Ho VP, Roach MJ, Berg KA (2023). In their own words: recovery after emergency general surgery in the older patient. Trauma Surg Acute Care Open.

[29] Hellstadius Y, Malmström M, Lagergren P (2019). Reflecting a crisis reaction: Narratives from patients with oesophageal cancer about the first 6 months after diagnosis and surgery. Nurs Open.

[30] Diehl M, Hay EL (2013). Personality-Related Risk and Resilience Factors in Coping with Daily Stress among Adult Cancer Patients. Res Hum Dev.

[31] Bahcecioglu Turan G, Özer Z, YanmıŞ S (2024). The effects of spiritual wellbeing on life satisfaction in hematologic cancer patients aged 65 and older in Turkey: mediating role of hope. Psychogeriatrics.

[32] Koenig HG (2012). Religion, spirituality, and health: the research and clinical implications. ISRN Psychiatry.

[33] Hamilton JB (2019). Emotional Suffering and the Use of Spirituality as a Resource among African American Cancer Survivors. J Cancer Educ.

[34] Balducci L (2019). Geriatric Oncology, Spirituality, and Palliative Care. J Pain Symptom Manage.

[35] Firkins JL, Tomic I, Hansen L (2025). Association of spirituality and quality of life in cancer survivors: a systematic review and meta-analysis. Support Care Cancer.

[36] Ludvigsson JF, Bergman D, Lundgren CI (2025). The healthcare system in Sweden. Eur J Epidemiol.

[37] Association WM, Declaration of Helsinki Ethical principles for medical research involving human subjects. https://www.wma.net/policies-post/wma-declaration-of-helsinki/.

