# Supplementary material for: Older adults’ experiences of adapting to changes after colorectal cancer surgery in Sweden: a qualitative study
Source: BMJ Open. 2026 Oct 12;16(10):e124929. doi: 10.1136/bmjopen-2026-124929 (PMC13662033; doi:10.1136/bmjopen-2026-124929)
Supplement: Supplementary file 2 [file bmjopen-2026-124929.reviewer_comments.pdf]

PEER REVIEW HISTORY

BMJ Open publishes all reviews undertaken for accepted manuscripts. Reviewers are asked to complete a checklist review form and are provided with free text boxes to elaborate on their assessment. These free text comments are reproduced below.

ARTICLE DETAILS

Title (Provisional)

Older adults' experiences of adapting to changes after colorectal cancer surgery in Sweden: A qualitative study

Authors

Amirpour, Anahita; Holmgren, Amina Guenna; Nilsson, Ulrica; Eckerblad, Jeanette

---

VERSION 1 - REVIEW

**Reviewer**	**1**
**Name**	**Lau, Jerrald**
**Affiliation**	**National University of Singapore, Saw Swee Hock School of Public Health**
**Date**	**26-Jul-2026**
**COI**	**None**

This was a succinctly written qualitative exploration on the impact of CRC on the lived experiences of older adults in Sweden. I just have some minor comments regarding the Methods for the authors' consideration, which I hope will help strengthen the manuscript:

Methods

1. (Data analysis, page 7) What was the role of author AGH in the analysis? From what I can tell of this subsection and the next, authors AA and JE were the ones who handled most of the thematic analysis process, but page 8 suggests that "all authors" were involved in reviewing and refining codes etc. This should be elaborated on for the sake of replicability.

2. (Rigor and reflexivity, page 8) Consider including a kappa statistic for agreement between the two independent coders.

3. (Rigor and reflexivity, page 8) This section could be further strengthened with a brief commentary on how the interviewer's (and other coders') personal/professional background could have biased the qualitative inquiry and inductive analysis.

**Reviewer**	**2**
**Name**	**Horn, Christopher**

**Affiliation**	**Cancer Institute New South Wales, Bowel Cancer Screening Program**
**Date**	**07-Aug-2026**
**COI**	**None**

Thank you to the authors for your work on this piece, in the most part it reads well and adds to the body of knowledge.

This qualitative retrospective cohort study study of short term recovery from CRC treatment in older adults (60+) was conducted alongside larger feasibility trial of evaluation tests.

It used coded interview samples rather than validated tools of assessing distress, functionality, or quality of life and interviewed 13 participants.

Limitations

This was an acknowledgement of it being a small cohort of 13 participants.

Also important, is the noted limitation in the demography of the sample –all Swedish speaking, educated, from within a limited geographic region and in a country with socialised medicine. This has a direct impact on the experiences which are reported, given how frequently travel and financial toxicity are reported in many cancer populations.

Despite the limitations, the context is still relevant a number of other communities (e.g. Australia and particularly the UK-NHS).

The short window of this study after treatment intervention sets it aside from the bulk of the published literature, and the timeframe adds some colour to our understanding - yet it is not entirely novel.

There are a number of items which could be improved or clarified:

• Rigour and reflexivity - differences in coding discussed until consensus – why was there not an independent adjudication?

• Given the stigma and perception around colostomy bags (acknowledging that the small sample size may prohibit this), it could be insightful to understand how the perception and adjustment varied between a stoma and non-stoma cohorts

• Flagging a possible translation issue, whereby some of the questions seem to be at odds with convention. E.g. Q4 and Q5 ask that if memory of attention were affected after surgery 'How did it happen?'. One assumes that a patient will have limited ability to meaningfully describe the physiological mechanism - which is what this question seems to ask. Suggest revisiting the translation and seeking clarification as to whether the question may have been closer to 'how did you experience this?' or 'in what way did this present?'

• The structure of the results, particularly the second grouping 'Fragility of life and presence of death' sits uncomfortably as this is essentially content of the first sub heading. Some themes e.g. fragility of life are touched on several times. Consider is there is opportunity to re-name the groups sub headings – e.g. extrinsic adaptations and intrinsic adaptations, to reduce duplication/fragmentation.

VERSION 1 - AUTHOR RESPONSE

Point by point responses

**Reviewer #1**	Thank you for your careful review and valuable suggestions, which helps us strengthen the manuscript and clarify several aspects of the study.
1. (Data analysis, page 7) What was the role of author AGH in the analysis? From what I can tell of this subsection and the next, authors AA and JE were the ones who handled most of the thematic analysis process, but page 8 suggests that "all authors" were involved in reviewing and refining codes etc. This should be elaborated on for the sake of replicability.	We have clarified the role of author AGH.
2. (Rigor and reflexivity, page 8) Consider including a kappa statistic for agreement between the two independent coders	We have revised the Rigor and reflexivity section to clarify the purpose of involving several researchers in the coding process. The researchers coded the transcripts separately to allow each researcher to engage with and interpret the data before bringing their perspectives to analytic discussions. The purpose was to include different perspectives and critically reflect on interpretations, and not to establish inter-coder agreement. Therefore, a kappa statistic was not considered appropriate for our analytic approach.
3. (Rigor and reflexivity, page 8) This section could be further strengthened with a brief commentary on how the interviewer's (and other coders') personal/professional background could have biased the qualitative inquiry and inductive analysis.	We have expanded upon this in the manuscript.
**Reviewer #2**	Thank you for your thoughtful and constructive comments, which helps us improve the clarity and methodological transparency of the manuscript

1. Rigour and reflexivity - differences in coding discussed until consensus – why was there not an independent adjudication?	We have clarified the Rigor and reflexivity section in the manuscript.
2. Given the stigma and perception around colostomy bags (acknowledging that the small sample size may prohibit this), it could be insightful to understand how the perception and adjustment varied between a stoma and non-stoma cohorts	A good point. However, only one participant received a temporary stoma, which did not provide sufficient data for a meaningful discussion between participants with and without a stoma.
3. Flagging a possible translation issue, whereby some of the questions seem to be at odds with convention. E.g. Q4 and Q5 ask that if memory of attention were affected after surgery 'How did it happen?'. One assumes that a patient will have limited ability to meaningfully describe the physiological mechanism - which is what this question seems to ask. Suggest revisiting the translation and seeking clarification as to whether the question may have been closer to 'how did you experience this?' or 'in what way did this present?	We have updated the interview guide, since this was an example of a follow-up question.
4. The structure of the results, particularly the second grouping 'Fragility of life and presence of death' sits uncomfortably as this is essentially content of the first sub heading. Some themes e.g. fragility of life are touched on several times. Consider is there is opportunity to re-name the groups sub headings – e.g. extrinsic adaptations and intrinsic adaptations, to reduce duplication/fragmentation	Thank you for this comment and we agree with your point. We have changed this theme to "Intrinsic Adaptation Through Confronting Mortality"

VERSION 2 - REVIEW

Reviewer 1

Name Lau, Jerrald

Affiliation National University of Singapore, Saw Swee Hock School of Public Health

Date 19-Sep-2026

COI

Thank you for addressing my comments. I have nothing more to add and wish the authors all the best in their research.

Reviewer **2**

Name **Horn, Christopher**

Affiliation **Cancer Institute New South Wales, Bowel Cancer Screening Program**

Date **22-Sep-2026**

COI

Thank you
