# Supplementary material for: Older adults’ experiences of adapting to changes after colorectal cancer surgery in Sweden: a qualitative study
Source: BMJ Open. 2026 Oct 12;16(10):e124929. doi: 10.1136/bmjopen-2026-124929 (PMC13662033; doi:10.1136/bmjopen-2026-124929)
Supplement: Supplementary file 3 [file bmjopen-16-10-s001.docx]

# **Interview guide**

**Q1.** Can you describe how you have been since you were discharged from the hospital?

**Q2.** Has anything been troubling or bothering you?
    If yes, what has been the most troubling for you?

**Q3.** How have you managed with daily activities? For example: using a mobile phone or computer, doing laundry, grocery shopping, transportation (driving or using public transport), cooking, managing medications, household cleaning, and finances.

**Q4.** Do you feel that your memory has been affected in any way after your surgery?
    If yes, can you give an example of how your memory has been affected after the surgery?

**Q5.** Do you feel that your attention has been affected in any way after your surgery?
    If yes, can you give an example of how your attention has been affected after the surgery?

**Q6.** Do you feel that your ability to concentrate has been affected in any way after your surgery? If yes, can you give an example of how your concentration has been affected after the surgery?

**Q7.** How would you describe your mood since the surgery?
    If you have noticed changes, in what way? Can you give an example?
    If not, have your relatives noticed any mood changes or similar changes?

**Q8**. How have you experienced taking initiative in daily life? Have you noticed any changes after your surgery? If yes, in what way? Can you give an example? When you plan to do something, does it usually get done?

**Q9.** Have you experienced any setbacks after the surgery? Have you encountered any difficulties or situations that did not go as planned? If yes, what kind? Can you give an example of how you handled it?

**Examples of follow-up questions**

- You mentioned… Could you tell me more about that?
- What do you mean by…?
- How would you describe…?
- Could you further elaborate…?

**Closing question**

**Q10.** Is there anything else related to this that you would like to share with me?
